# Heart rate variability and cold-induced vascular dilation after stimulation of two different areas of the ear: a prospective, single-blinded, randomized crossover study

**DOI:** 10.1186/s12906-024-04392-7

**Published:** 2024-02-13

**Authors:** Emmanuel Sagui, Damien Claverie, Wahiba Bidaut, Laurent Grelot

**Affiliations:** 1 European Hospital of Marseille, 13002 Marseille, France; 2 French Biomedical Research Institute, 91220 Bretigny sur Orge, France; 3 Institute of Technology, Aix-Marseille University, dept HSE, 13708 La Ciotat, France; 4 French Military Hospital Laveran, 13384 Marseille, France

**Keywords:** Noninvasive vagus nerve stimulation, Autonomic nervous system, Heart rate variability (HRV), Auricular neuromodulation, Cold-induced vasodilation (CIVD), Auricular acupuncture, Auriculotherapy

## Abstract

**Background:**

Both noninvasive transauricular vagus nerve stimulation (taVNS) and traditional medical practice (TMP), such as auriculotherapy, use the auricle as a starting point for stimulation, but with two different conceptual frameworks: taVNS depends on vagal afferences to account for its effects, whereas TMP requires stimulation of the ear with high topographical accuracy regardless of the afferent nerves. The aim of this study was to measure heart rate variability (HRV) and cold water–induced vasodilation (CIVD) after puncturing two different ear points with the same afference but that should have opposite effects according to TMP.

**Methods:**

Ten healthy subjects were investigated in this single-blinded crossover study over three sessions. In the first session, sympathetic activation was performed via cold water immersion of the right hand, with recordings taken from multiple fingers. HRV was assessed in the time domain (square root of the mean squared differences of NN intervals (RMSSD)) and frequency domain (low (LF) and high frequencies (HF)). In the second and third sessions, the same skin immersion test was performed, and mechanical stimulation was applied to the ear at two different points on the internal surface of the antitragus, one with alleged parasympathetic activity and the other with alleged sympathetic activity. The stimulation was done with semipermanent needles.

**Results:**

Stimulation of the point with alleged parasympathetic activity immediately resulted in a significant decrease in RMSSD in 75% of the subjects and in LF in 50% of the subjects, while stimulation of the point with alleged sympathetic activity resulted in an increase in HF and RMSSD in 50% of the subjects. Stimulation of these points did not affect the CIVD reflex. The 20 min cold water immersion induced an immediate decrease in LF and the LF/HF ratio and an increase in HF. The skin temperature of the nonimmersed medius significantly decreased when the contralateral hand was immersed, from 34.4 °C to 31.8 °C.

**Conclusions:**

Stimulation of two different ear points innervated by the same afferent nerves elicited different HRV responses, suggesting somatotopy and a vagal effect beyond vagal afferences. These results are not in accordance with the claims of TMP.

**Trial registration:**

NCT04130893 (18/10/2019) clinicaltrials.com.

## Introduction

Invasive peripheral nerve stimulation, most often through the vagus nerve, has proven to be an effective therapeutic option for treating drug-refractory illnesses such as epilepsy and depression [1, 2].

However, the widespread adoption of this technique was hampered by the invasive procedure performed by a few teams, the cost of the devices and the high rate (as high as 19%) of device-related side effects, limiting its use to a few highly selected patients [3, 4].

To overcome these limitations, noninvasive devices have been developed. Although several nerves, such as the occipital nerve and the trigeminal nerve, have been targeted for noninvasive electrical stimulation, the vagus nerve has been the most studied [5–7]. The vagus nerve benefits from invasive stimulation studies and can be stimulated easily by noninvasive devices either on the ear via the auricular branch or on the neck via the cervical branch [8]. Electrical stimulation of the auricular branch activates the nucleus tractus solitari and central vagal projections such as the dorsal raphe, the locus coeruleus, the hippocampus and the hypothalamus according to fMRI studies [9–11]. Central activation via a neuromodulation mechanism could account for clinical benefits [12].

The indications for electrical noninvasive vagus nerve stimulation (niVNS) outweigh those for invasive VNS (iVNS) because of the greater incidence of minor adverse effects, lower cost and easier implementation. niVNS is used for depression [13, 14] and epilepsy [15–17], but the level of evidence for niVNS is lower than that for iVNS [18, 19]. Data suggest modest beneficial effects of niVNS for headaches [5], pain control [16] and cognitive impairment [20], but these effects need further investigation.

Not only electrical niVNS but also mechanical niVNS has been performed without initial knowledge of vagal nerve involvement in some traditional medical practices (TMPs), especially when stimulating the ear. In ancient times, treatment of sciatic pain or toothache by ear cauterization or scarification was reported in Europe, whereas some ear acupuncture points have been described in traditional Chinese medicine [21]. The two systems merged substantially after the publication of the 20th century tribute by Nogier, who was the first to describe a somatotopy in the ear and to expand indications beyond pain relief, coining this TMP “auriculotherapy” [22]. Even if there are some minor differences between TMPs, technicians need to master one to two hundred stimulating points and stimulate them with topographical accuracy [23, 24]. However, few points have been investigated scientifically [25, 26], and most of the connections between stimulating points and their presumed targets are based on empirical data and expert consensuses [23, 24].

Conversely, the modern conception of neurostimulation does not require somatotopy but rather nerve afferences, namely, the great occipital nerve, the auriculo-temporal nerve and the vagus nerve in the ear. In this conception, stimulation points are moved down to stimulate the corresponding nerve afferences, for example, cymba conchae for the vagal nerve [27, 28].

The aim of this study was to measure the physiological consequences of two different points that originate from the same afferent nervous area but have opposite effects according to TMP. Similar physiological effects would support the validity of global afferent nerve stimulation, such as that already performed by niVNS devices, while different physiological effects would suggest the legitimacy of the topographic accuracy described by TMP.

## Patients and methods

This prospective, single-blinded, crossover study took place in a laboratory room of the Department of Cardiovascular Investigations at the European Hospital of Marseille, France, from March 2019 to February 2020.

### Subjects

Because the COVID-19 pandemic disrupted the local research team, 14 out of the 24 planned healthy subjects volunteered for the study. Volunteers were recruited via advertisement at the European Hospital of Marseille. The inclusion criteria were as follows: male or female aged 18–60 without experience with auriculotherapy or ear acupuncture, with a body mass index ≤ 30 and no concomitant participation in another research study. The exclusion criteria were as follows: previous experience with auriculotherapy; current medication; history of diabetes, atrial fibrillation, hypertension, syncope or palpitations; body mass index > 30; nonremovable ear modification, such as piercings; consumption of psychoactive substances; excessive amounts of alcohol (over two units per day for regular use or six units per day for recreational use); or tobacco consumption at over 5 cigarettes per day. Patients were required to refrain from caffeine and tobacco for at least 4 h before each visit.

### Design of the study

The study was designed to test two different stimulation points taken from acupunctural layouts. The first point was situated on the posterior area of the internal surface of the antitragus, on the G15 location according to the World Federation of Chinese Medicine Societies (WFCMS) [24], or on the CW2 area described by Oleson [24, 29]. This point is said to have the strongest parasympathetic activity [30].

The second point was on the anterior area of the internal surface of the antitragus on the G13 (WFCMS) or CW3 location (Oleson). This point is said to have the strongest sympathetic activity [30]. Both points were in the AT4 area according to the World Federation of Acupuncture-Moxibustion Societies (WFAS) [23, 24].

According to the TMP theoretical framework, stimulation of an ear point “regulates” disequilibrium either by regulating “energy”, namely, “Qi”, or by returning to a previous steady state [22, 29, 30]. According to this framework, it is not possible to elicit a sympathetic or parasympathetic response simply by stimulating an ear point. Hence, an autonomic imbalance should be performed before eliciting a parasympathetic or sympathetic response. Hence, sympathetic stimulation was performed before ear stimulation to sensitize the patients to the outcome measures. The sympathetic stimulation consisted of cold water immersion of the hand before ear stimulation, as described below.

**Fig. 1:**
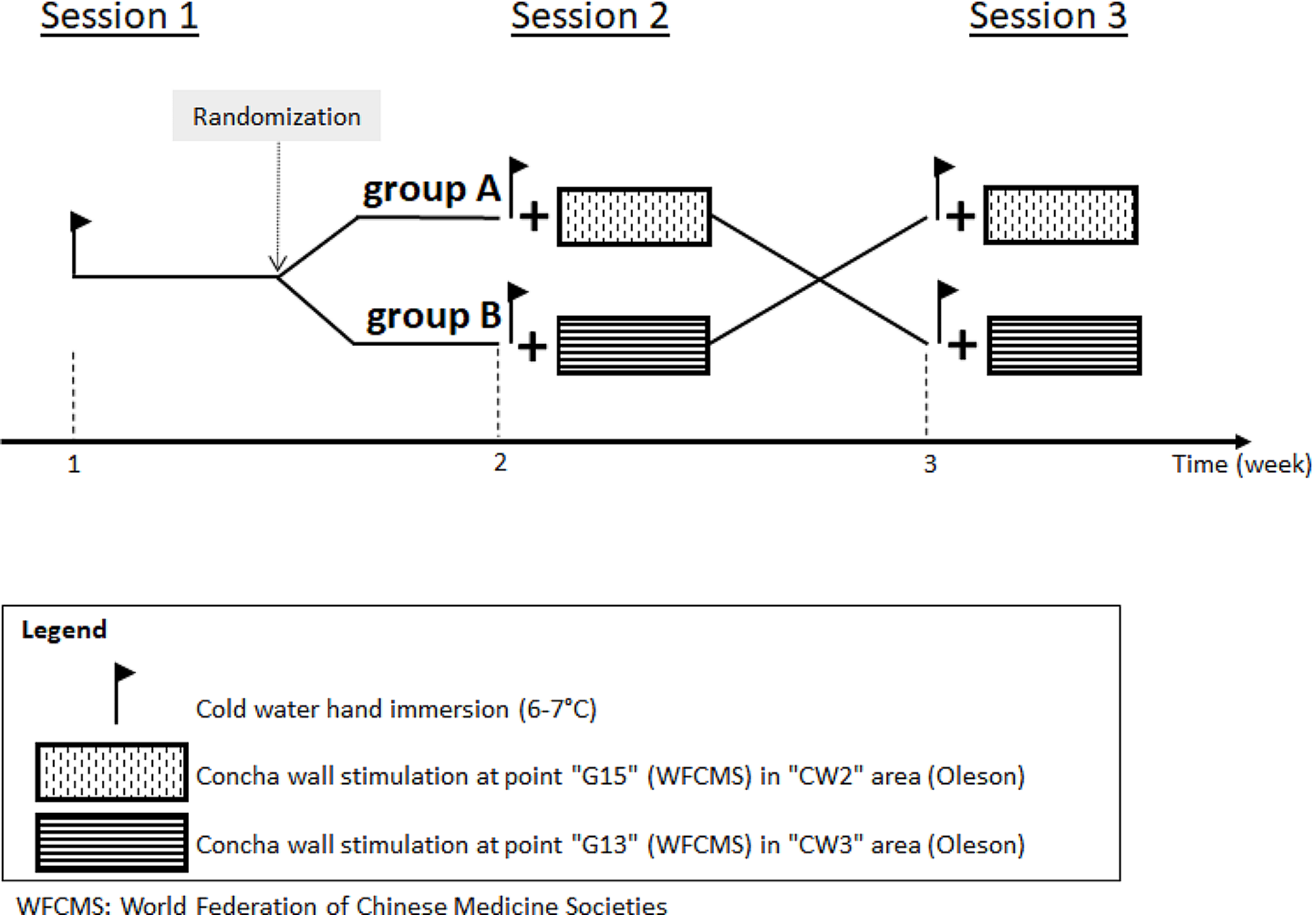
Study design. WFCMS: World Federation of Chinese and Moxibustion Societies

The study was done in three sessions, with intervals of at least one week between sessions, as outlined in Fig. 1. The first session tested tolerance to a 20 min cold water immersion of the hand without ear stimulation. The next two sessions took place in a randomized order with a block size of 6 according to a computerized procedure [31]. Group A underwent G15/CW2 stimulation in the second session and G13/CW3 stimulation in the third session. Group B underwent G13/CW3 stimulation in the second session and G15/CW2 stimulation in the third session. The subjects were allocated to group A or B by the main investigator (ES) according to the prespecified randomization list before the second session. The subjects were blinded to the stimulation.

### Sympathetic stimulation procedure

Cold water immersion in the extremities elicits strong sympathetically induced vasoconstriction, leading to a rapid decrease in limb temperature [32]. This phenomenon can be overcome by a paradoxical and temporary vasodilation process called “cold-induced vasodilation” (CIVD), which occurs 5–10 min after cold exposure and allows partial rewarming [33]. CIVD is thought to be caused by a sudden drop in sympathetic activity. Because peripheral vasoconstriction or vasodilation is mediated only by sympathetic nerves and accounts for local skin temperature, the measure of local skin temperature is a good surrogate marker for sympathetic activity [33].

Cold water immersion was performed by placing the subject’s left hand in a 6–7 °C regulated water bath at 6–7 °C for 20 min. During hand immersion, the subject was asked not to move on the chair and not to shake the fingers of the immersed hand.

### Ear stimulation procedure

To comply with the spatial resolution requirements, the ear was mechanically stimulated with semipermanent stainless-steel needles 3.7 mm in length by 0.5 mm in diameter (ASP®, Sedatelec, Irigny, France). Their placement was controlled via a plastic introducer that allowed insertion at a constant depth. No electrical, magnetic or mechanical stimulation was applied to the needle after its placement.

Both ears were punctured in the same order for all patients, the right ear first. The stimulus lasted 30 min.

### Variables recorded

#### Heart rate variability

Heart rate was measured using an Actiwave device (CamNtech Ltd., Cambridgeshire, UK). After skin preparation using an abrasive paste, the cardio unit of the device was placed between V1 and V2, and the second electrode was placed between V4 and V5. The electrocardiogram signal was sampled at 1024 Hz with a 10-bit resolution.

Heart rate variability (HRV) was analyzed using Kubios HRV Premium software v3.2.0 [34]. Time domain and frequency domain methods were used. Time domain methods included the standard deviation of the NN intervals (SDNN) and the square root of the mean squared difference of successive NN intervals (RMSSD), the latter being the most robust time domain estimate of vagal activity [35]. Frequency domain methods address the variance distribution as a function of frequency. Two bands were studied: the low-frequency (LF) component, ranging from 0.04 to 0.15 Hz, and the high-frequency (HF) component, ranging from 0.15 to 0.4 Hz [36]. LF represents a mixture of sympathetic and parasympathetic activity, whereas HF reflects parasympathetic stimulation. Both components were recorded via 5-minute segment analysis (epoch) and are reported in normalized units, which represent the relative value of each component in proportion to the total power minus the very low-frequency component, i.e., the power spectrum ranging from 0.0033 to 0.04 Hz. The ratio of LF to HF power was also assessed.

#### Sympathetic skin activity

**Fig. 2:**
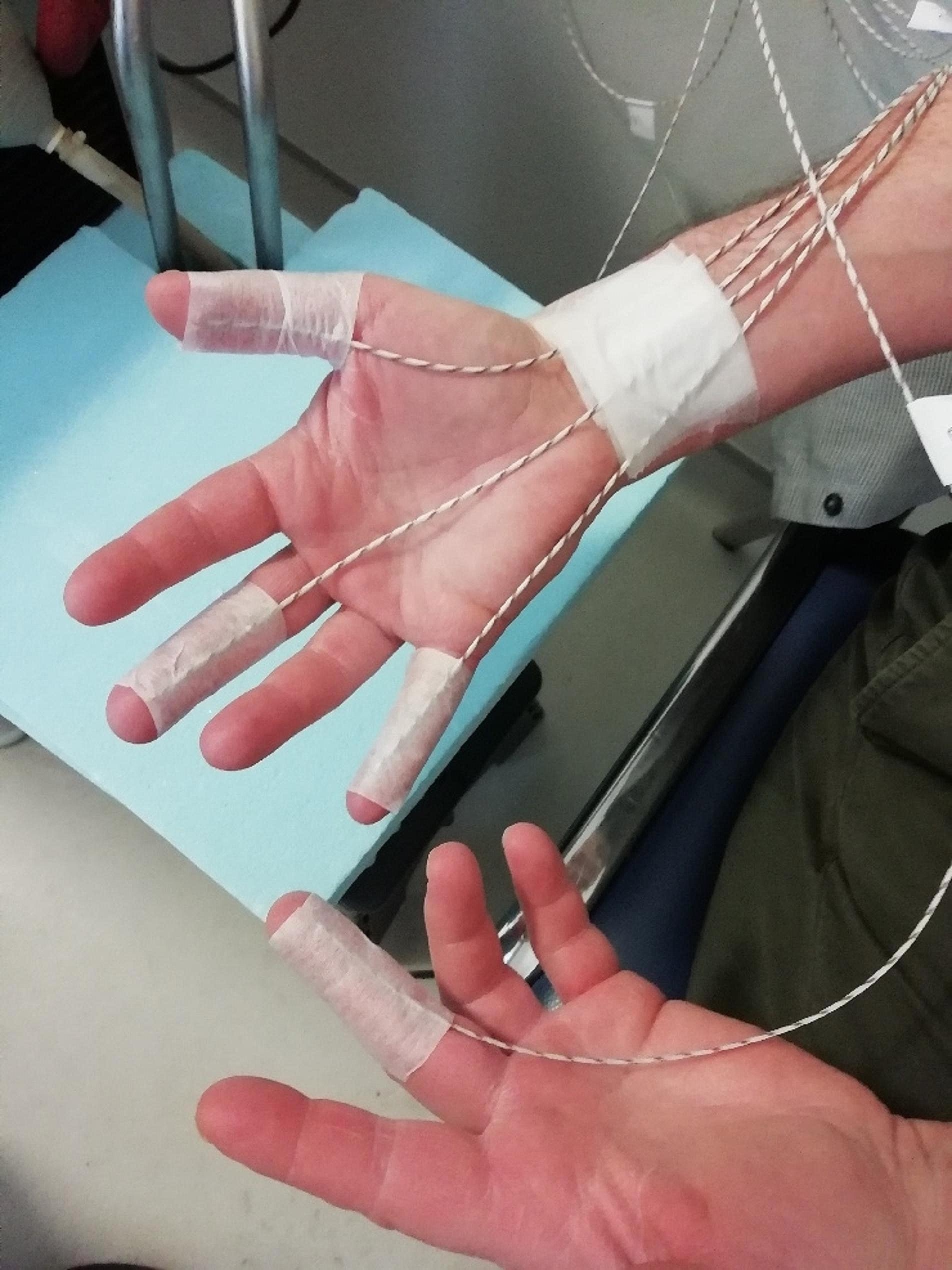
Installation of the thermocouples

Changes in sympathetic skin activity, assessed by changes in skin temperature, were monitored by thermocouples at a frequency sample of 1 Hz. Skin temperature was measured at five sites with thermocouples: the pads of the second phalanx of the thumb, the third phalanx of the third and fifth fingers and the wrist of the immersed hand, and the pad of the third phalanx of the third finger of the nonimmersed hand. Thermocouple positioning and fastening with single-ply adhesive tape were highly standardized. Figure 2 illustrates the installation of the thermocouples.

**Fig. 3:**
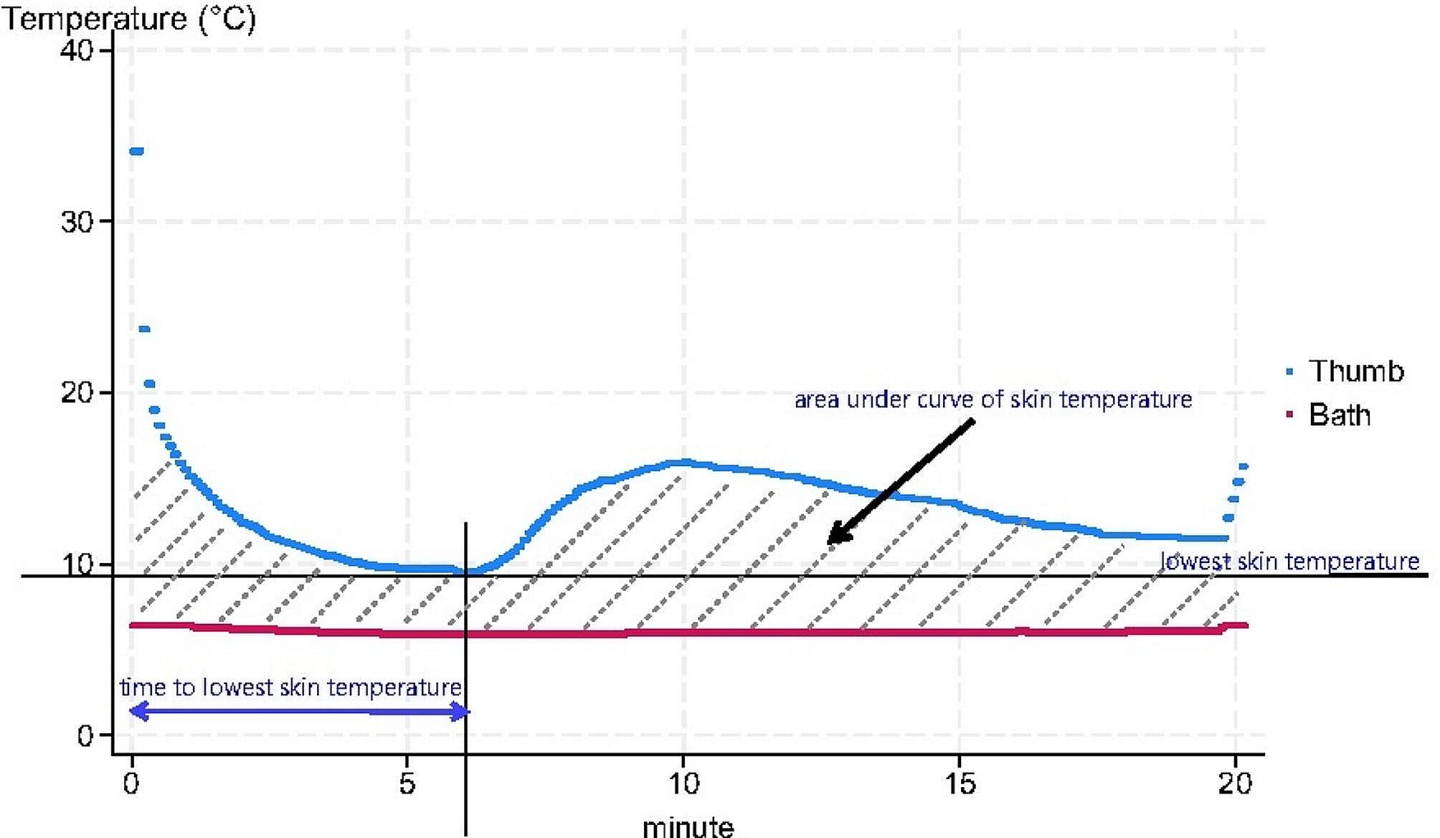
Variables recorded

The studied variables were the lowest skin temperature, the time from cold water immersion to the lowest temperature and the area under the curve (AUC) of skin temperature, calculated as the summation of all the differences between the bath temperature and skin temperature at the finger of interest during the first 1210 s. The lowest skin temperature was defined as the temperature at which the temperature was stable for at least 5 s. Figure 3 shows the variables recorded.

### Outcome measures

The primary outcome measure was the difference in the lowest skin temperature between the two interventions.

The secondary outcome measures were the time from cold water immersion to the lowest temperature, the AUC of skin temperature, RMSSD, LF, HF and the ratio of LF to HF power.

### Number of subjects

To compute the number of subjects needed, we hypothesized that the difference in the lowest skin temperature would be 2.39 ± 2.00 °C between the two interventions, with a type I error of 5% and a type II error of 20%. According to SAS V9.4 (SAS Institute, Inc., Cary, NC), 24 subjects were needed. With a dropout rate of 20%, 30 subjects were planned to be recruited.

### Experimental procedure

Sessions were scheduled in the afternoon hours from 14:00 to 17:00. All sessions took place in the same room, whose temperature was kept at 25 °C. Patients were required to restrain from caffeinated beverages at least 4 h before the session, from excessive physical activity or from smoking 48 h before the procedure and from eating at least two hours before.

First, the Actiwave® device was placed on the subject’s chest. The subject was required to sit comfortably in the experimental chair and to refrain from engaging in any physical activity for 5 min.

**Fig. 4:**
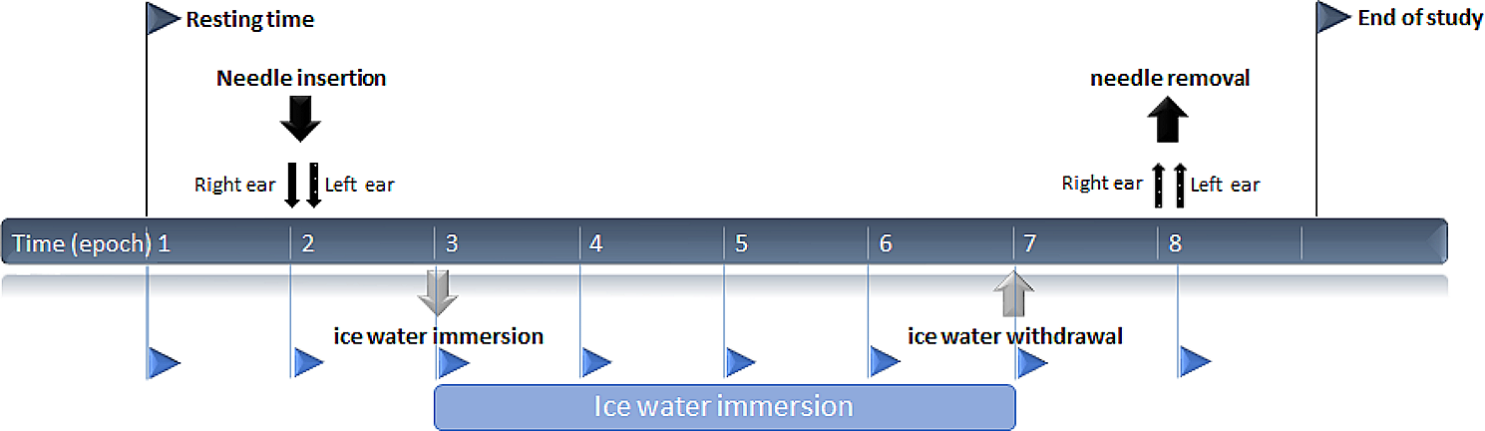
Experimental procedure

Then, the thermocouples were positioned on the fingers and the wrist as described above. Figure 4 summarizes the experimental procedure. After a five-minute rest, the subject underwent needle insertion in both ears. After 5 more minutes, the subject was required to immerse his left hand in cold water for 20 min. The needle was removed five minutes after hand removal. In the first session, no needle was inserted. Because the removal of the steel needles could take up to 30 s, the last epoch started after the removal of the second needle.

### Statistical analysis

Statistical analysis was performed using Stata 18 (StataCorp LLC, College Station, Texas, USA). Quantitative variables are reported as the median with interquartile range (IQR) rather than the mean and standard deviation because their distributions were not Gaussian. Categorical variables were compared between two groups using the chi-squared test with the Mantel–Haenszel correction. Quantitative variables were analyzed using the Kruskal–Wallis test. Two nonindependent quantitative variables were compared using Kendall’s test.

### Ethics and regulation

All the requirements of French law were fulfilled. This study was approved by an ethics committee on 26/10/2018 (the Ouest IV ethics committee) under reference 2018-A02784-51 and was registered on 18/10/2019 at clinicaltrials.com under reference NCT04130893. Informed consent was obtained from all subjects prior to their participation.

### Data availability

The datasets used and analyzed during the current study are available from the corresponding author on reasonable request.

## Results

### Demographic data

Fourteen subjects volunteered for the study. One patient was excluded before the first session because arrhythmia was diagnosed from the EKG before the study. One subject was excluded at the end of the first session because no CIVD was elicited. Two additional subjects withdrew from the study after the end of the first session, the first because of a house move and the second because cold water immersion was not bearable. Hence, analysis was carried out on the ten remaining subjects (2 male and 8 female subjects, aged 20–57 years, mean age 41 ± 13.5 years). Four subjects were assigned to group B, and 6 subjects were assigned to group A. All subjects but one were right-handed. The demographic characteristics were comparable between the two groups (Table 1).

**Table 1:** Patient characteristics at baseline

	Group A (*N* = 6)	Group B (*N* = 4)
Age (median (range))	47 (28–50)	28 (27–49)
Women (n,%)	4 (67%)	3 (75%)

Regardless of the session modality, the ambient temperature ranged from 25.2 ± 1.2 °C at the beginning of the session to 25.4 ± 1.1 °C at the end of the session. The cold water temperature was 6.1 ± 0.5 °C at the beginning of the session and 7.1 ± 2.7 °C at the end of the session.

### Effects of cold water immersion

#### Cold water immersion and HRV

Cold water immersion resulted in an immediate decrease in LF and the LF/HF ratio and an increase in HF in all but one subject. During the 20 min of cold water immersion, LF and the LF/HF ratio increased either in the first five minutes of immersion or after 15 min of immersion. One subject had an initial increase in LF and LF/HF but later exhibited a steep and continuous decrease in LF and LF/HF without rebound.

No trend was observed in RMSSD or SDNN during cold water immersion. RMSSD increased in 2 subjects, remained unchanged in 1 subject and decreased in 3 of them. SDNN increased in 3 subjects and decreased in 3 of them. HRV data for the other subjects of the study were not available for technical reasons. On cold water withdrawal, RMSSD increased in 5/6 subjects (83%), and SDNN increased in 4/6 subjects (67%).

#### Cold water immersion and CIVD

**Fig. 5:**
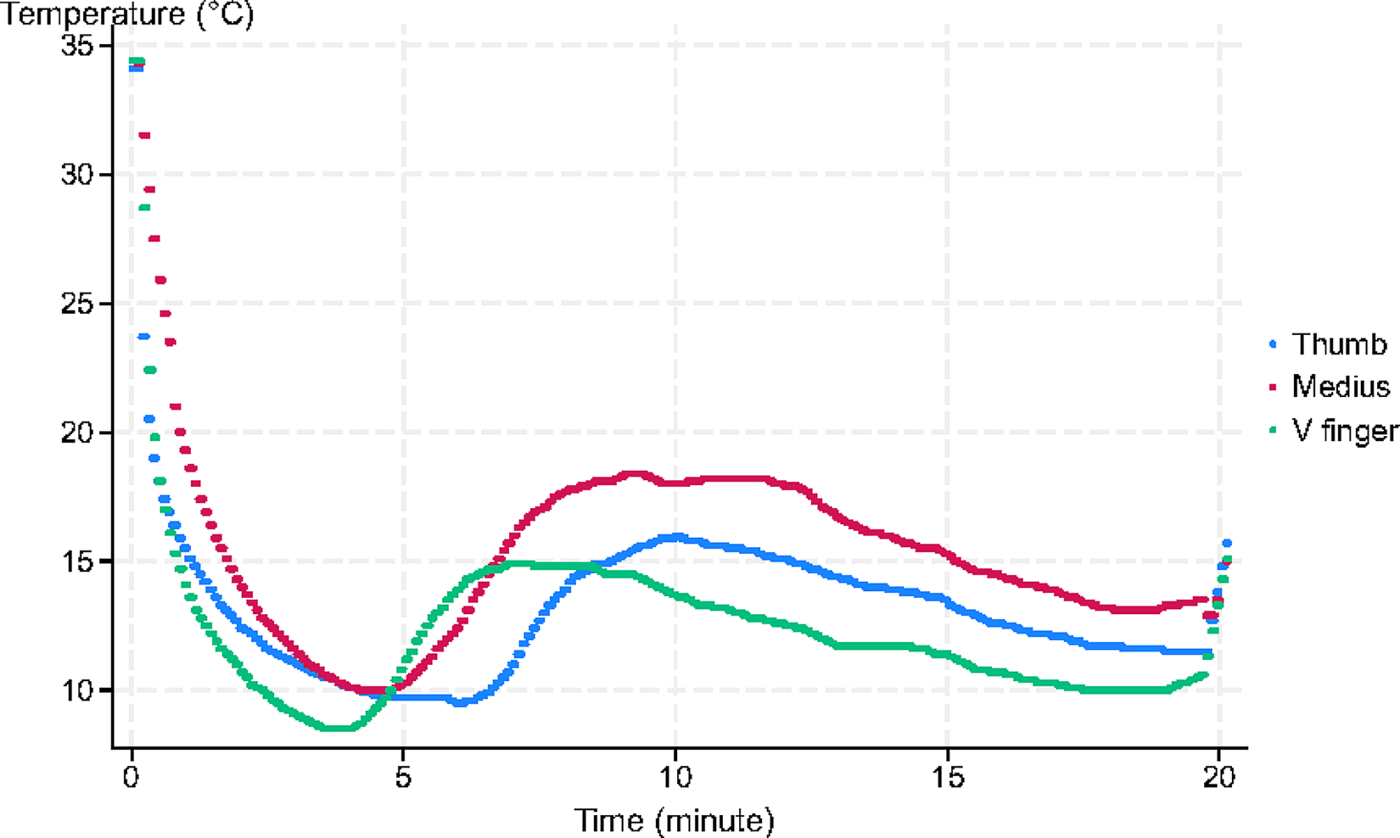
Evidence of a cold-induced vasodilation reflex

All the participants exhibited CIVD in at least one finger of the immersed hand, as illustrated in Fig. 5, but two patients exhibited CIVD in one of three fingers only.

The skin temperature ranged from 5.3°C to 12°C on the immersed thumb and from 5.3°C to 15.9°C on the immersed medius. The lowest values were observed throughout the immersion, from 5’06’’ to 19’38’’, the latter indicating no CIVD in the recorded finger.

The wrist temperature decreased to 13.4 °C (IQR 11.9 to 15.3), without any significant differences throughout the sessions. No CIVD was recorded in the immersed wrist regardless of the session.

The skin temperature of the nonimmersed medius significantly decreased when the contralateral hand was immersed, from 34.4 °C (IQR 33.9 to 35.1) to 31.8 °C (IQR 29.6 to 32.8) (*p* < 0.01). This decrease was similar between the sessions.

### Effect of G15/CW2 stimulation (alleged parasympathetic activity)

#### G15/CW2 stimulation and HRV

Needle insertion resulted in an increase in the LF/HF ratio and LF and a decrease in HF in 4 subjects; the opposite response occurred in 2 subjects, and no change in HRV occurred in 2 subjects. RMSSD and SDNN also decreased in all but 2 subjects, who had an increase in these 2 parameters.

Immediate cold water immersion induced a decrease in LF and the LF/HF ratio and an increase in HF in 6 subjects; the opposite response occurred in one subject, and no response occurred in one subject. No trend was observed in RMSSD or SDNN. The raw values for the RMSSD and LF/HF ratio are displayed in Tables 2 and 3, respectively.

**Fig. 6:**
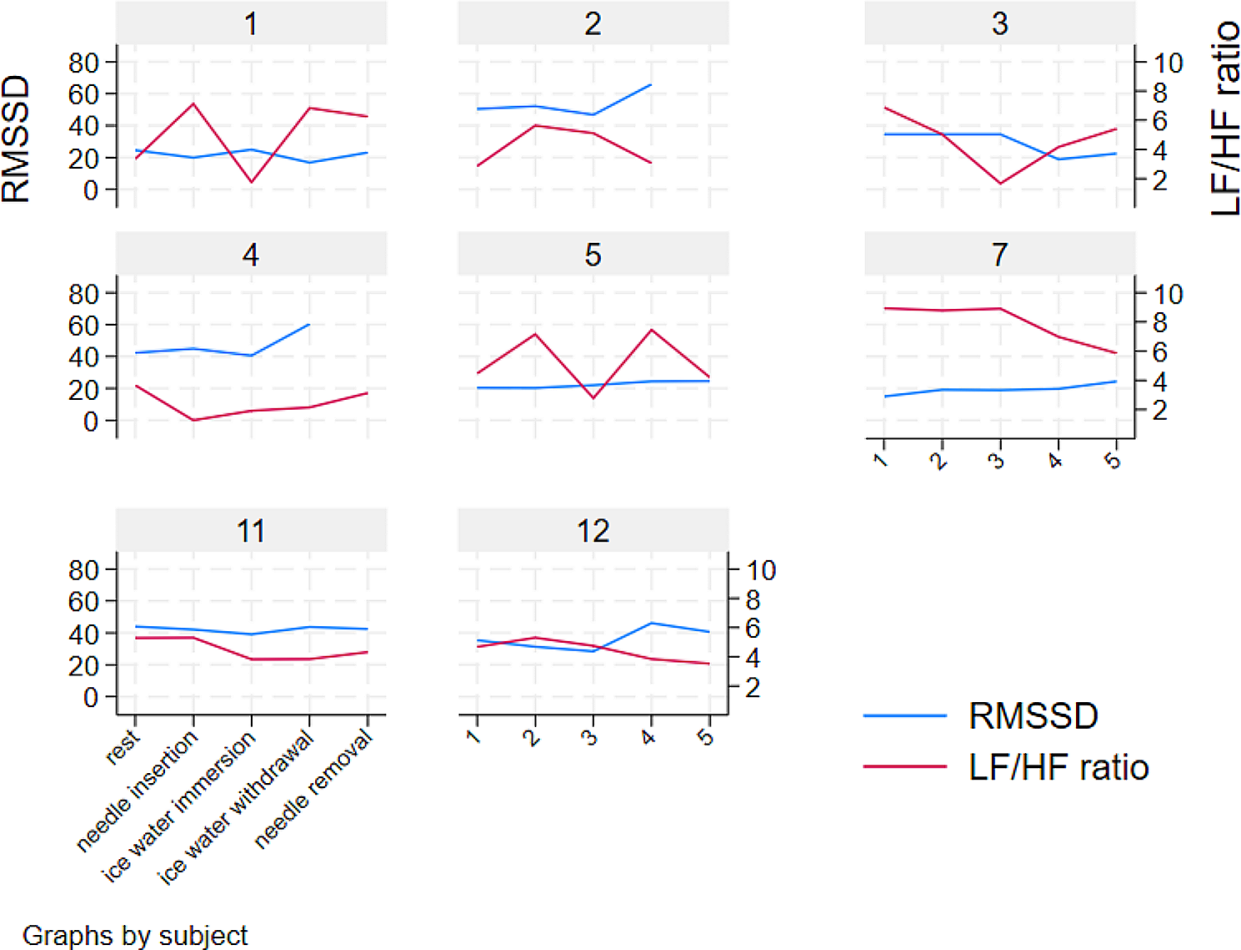
HRV and LF/HF ratio changes for all subjects after G15/CW2 stimulation (alleged parasympathetic activity)

After 20 min of hand immersion in cold water, the LF/HF, LF and HF ratios were similar to their values immediately after immersion (*p* = 0.44, *p* = 0.44 and *p* = 0.46, respectively); however, the RMSSD and SDNN decreased in half of the subjects. Figure 6 illustrates the changes in the LF/HF ratio and RMSSD for all subjects.

The removal of the needle was not associated with any significant changes in the LF/HF ratio, LF or HF (*p* = 0.15, *p* = 0.17 and *p* = 0.81, respectively), but the RMSSD and SDNN increased in all but two subjects.

**Table 2:** Square root of the mean squared differences in successive NN intervals (RMSSD) at key epochs after G15/CW2 stimulation (alleged parasympathetic activity)

RMSSD			Epochs		
subject	rest	needle insertion	cold water immersion	cold water withdrawal	needle removal
1	24.6	19.9	25.0	16.8	23.1
2	49.7	46.3	44.5	48.4	69.7
3	34.5	34.5	34.5	18.8	22.5
4	29.0	30.4	41.6	46.9	45.6
5	20.4	20.3	22.0	24.4	24.6
7	15.0	19.2	18.9	19.7	24.4
11	49.5	40.4	37.3	50.8	50.0
12	22.5	20.2	18.0	28.0	32.5
p value	<- 0.06 ->		<- 0.16 ->		
		<- 0.08 ->		<- 0.06 ->	

**Table 3:** Low-frequency/high-frequency ratio (LF/HF ratio) after G15/CW2 stimulation (alleged parasympathetic activity)

LF/HF ratio			Epoch		
subject	rest	needle insertion	cold water immersion	cold water withdrawal	needle removal
1	3.3	7.1	1.8	6.8	6.3
2	3.2	4.4	5.7	3.8	3.5
3	6.9	5.0	1.7	4.2	5.4
4	5.1	3.6	3.8	3.1	3.3
5	4.5	7.2	2.8	7.5	4.2
7	8.9	8.8	8.9	7.0	5.9
11	3.2	2.1	1.7	2.0	2.7
12	4.5	6.1	5.2	2.8	1.7
p value	<- 0.38 ->		<- 0.44 ->		
		<- 0.17 ->		<- 0.15 ->	

#### G15/CW2 stimulation and CIVD

Stimulation at this point did not significantly change the minimal skin temperature, time to minimal skin temperature or area under the curve of skin temperature vs. bath temperature during the first 20 min at the thumb, the medius or the little finger. Table 4 reports the values for the thumb and the medius.

**Table 4:** Temperature and area under the curve during the 3 sessions on the immersed thumb and medius. AUC: area under the curve; T°: temperature; T°min: lowest skin temperature

	No ear stimulation	G15/CW2 ear stimulation (alleged parasympathetic activity)	G13/CW3 ear stimulation (alleged sympathetic activity)
**T°min**			
Thumb	7.7 [6.8–9.5] °C	8 [6.8–8.5] °C	7.9 [7.1–9.1] °C
Medius	7.4 [6.3–8.9] °C	7 [6.3–8.9] °C	8.1 [7–8.3] °C
**Time to T°min**			
Thumb	6’05’’ [5’28’’ – 15’46’’]	4’43’’ [4’21’’ – 8’58’’]	5’51’’ [3’41’’ – 5’56’’]
Medius	5’37 [4’38’’ – 14’10’’]	5’29’’ [4’37’’ – 6’26’’]	4’38’’ [3’53’’ – 5’29]
**AUC**			
Thumb	5589 [4167–8656]	5706 [4854–7223]	6103 [4999–6979]
Medius	5886 [4767–7904]	6210 [6003–7129]	5961 [5106–7589]

### Effect of G13/CW3 stimulation (alleged sympathetic activity)

#### G13/CW3 stimulation and HRV

Needle insertion resulted in a decrease in the LF/HF ratio and LF and an increase in HF for 4 subjects, the opposite response for 2 subjects, and no change in HRV for the remaining subjects. RMSSD and SDNN increased in half of the subjects, decreased in one subject and remained unchanged in the other subjects.

Immediate cold water immersion did not induce a trend of either a decrease or an increase in LF, HF or the LF/HF ratio. RMSSD and SDNN decreased in all patients but one (SDNN) or two (RMSSD). The raw values for the RMSSD and LF/HF ratio are displayed in Tables 5 and 6, respectively.

**Fig. 7:**
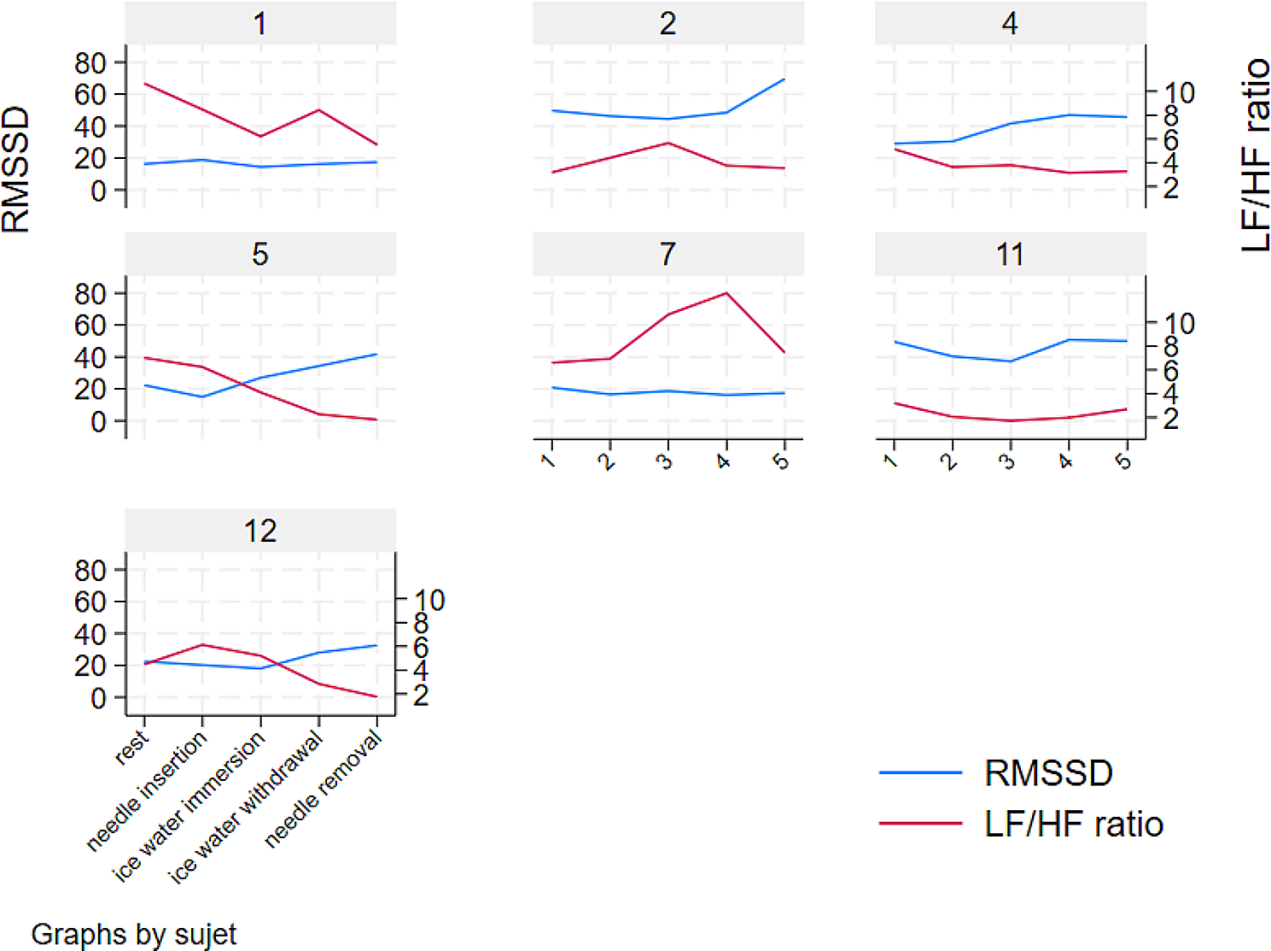
HRV and LF/HF ratio changes for all subjects after G13/CW3 stimulation (alleged sympathetic activity)

After 20 min of hand immersion in cold water, the LF/HF ratio, LF and HF did not change significantly from baseline (*p* = 0.12, *p* = 0.12 and *p* = 0.12, respectively), and no trend was observed for RMSSD or SDNN. Figure 7 illustrates the changes in the LF/HF ratio and RMSSD for all subjects.

The removal of the needle was not associated with any change in the LF/HF ratio, LF or HF (*p* = 0.20, *p* = 0.75 and *p* = 0 = 0.20, respectively), nor was any trend for RMSSD or SDNN.

**Table 5:** Square root of the mean squared differences in successive NN intervals (RMSSD) at key epochs after G13/CW3 stimulation (alleged sympathetic activity)

RMSSD			Epoch		
subject	rest	needle insertion	cold water immersion	cold water withdrawal	needle removal
1	16.2	18.8	14.4	16.2	17.4
2	50.4	52.2	46.8	65.9	
4	42.3	44.9	40.6	60.3	
5	22.3	15.0	27.0	34.3	41.8
7	20.8	16.6	18.7	16.2	17.4
11	44.0	42.1	39.1	43.7	42.5
12	35.4	31.3	28.5	46.2	40.6
p value	<- 0.09 ->		<- 0.07 ->		
		<- 0.08 ->		<- 0.17 ->	

**Table 6:** Low-frequency/high-frequency ratio (LF/HF ratio) after G13/CW3 stimulation (alleged sympathetic activity)

LF/HF ratio			Epoch		
subject	rest	needle insertion	cold water immersion	cold water withdrawal	needle removal
1	10.7	8.5	6.2	8.4	5.5
2	2.9	5.6	5.1	3.1	
4	3.7	1.3	1.9	2.2	3.1
5	7.0	6.2	4.1	2.3	1.8
7	6.6	6.9	10.7	12.5	7.4
11	5.3	5.3	3.8	3.8	4.3
12	4.7	5.3	4.7	3.8	3.5
p value	<- 0.10->		<- 0.08->		
		<- 0.09 ->		<- 0.08 ->	

#### G13/CW3 stimulation and CIVD

Stimulation at this point did not significantly change the minimal skin temperature, time to minimal skin temperature, or area under the curve of skin temperature vs. bath temperature during the first 20 min at the thumb, the medius or the little finger. The only significant difference was observed between the area under the curve of the temperature of the medius between G15/CW2 stimulation and G13/CW3 stimulation (*p* = 0.04). Table 1 reports the values for the thumb and the medius.

## Discussion

Stimulation of the inner part of the antitragus by semipermanent needles elicited two different responses of HRV, according to the site of stimulation. The stimulation of the G15/CW2 point, which is credited with parasympathetic activity, induced an immediate decrease in the HF component and RMSSD, indicating a decrease in parasympathetic activity. Conversely, stimulation of the G13/CW3 point, which is credited with sympathetic activity, induced an immediate decrease in the LF component, which reflects a mixture of sympathetic and parasympathetic activity but also an increase in the HF component, indicating an increase in parasympathetic activity.

This study illustrated that the stimulation of two different points that are 4 to 5 mm apart and innervated by the same nerve elicited two different HRV responses, but not in accordance with TMP claims.

In this study, stimulation at both points did not modify the sympathetic stimulation induced by immersion in a cold water bath, as HRV and CIVD did not significantly change. The only significant difference was observed between the AUC of the temperature of the immersed medius under the two stimulating conditions. However, given the multiple comparisons, the low number of subjects and the distribution of the two compared populations, this difference was not considered further. To date, no study has investigated the effect of auricular stimulation on CIVD.

Stimulation of the ear by an acupuncture needle in the left inferior concha at the “Lung 1” point induced significant parasympathetic activity during and 60 min after stimulation [37]. The stimulation of two different points (one in the triangular fossa at the “Shenmen” point and the other on the helix root at “point 0”) kept the ratio lower during the postoperative period after hemicolectomy [38].

This study was hampered by the low number of subjects because recruitment stopped during the COVID-19 pandemic, and the research team was disrupted afterward. This low number of subjects could have accounted for the high type 2 error.

The HRV data could have been biased by the following factors. First, time and frequency domain measurements decrease with age, especially between the second and third decades [39, 40]. In our study, age ranged from 20 to 57 years, but the number of subjects did not allow any adjustment for age. Second, sex was another potential bias, as women show parasympathetic dominance that induces a lower LH/HF ratio [41]. In our study, all but two of the subjects were women. The crossover design of the study may have overcome this potential bias, as the subjects were compared with each other, and changes in the outcome variables were measured. Another bias associated with women is that hormonal status was not monitored, as ovulatory cycles can affect HRV indices. Third, HRV analysis requires stationarity. The one-week interval between two sessions may have hampered this hypothesis, but the interval was chosen to sweep any residual effect of ear stimulation from one session to the other. A way to overcome some of these aforementioned biases would have been to use a nonlinear analysis instead of frequency analysis. For example, the use of the classification angle framework, a nonlinear analysis, does not require the assumption of signal stationarity and requires as little as 10 s of HRV data [42].

Finally, the standardized sitting position of the subjects could have biased the results compared with the supine position, but stimulation of the inferior concha with an acupuncture needle elicits parasympathetic activity in both positions [43].

## Conclusion

This study provides evidence that stimulation at two different points innervated by the same afferent nerves elicits either an increase or a decrease in parasympathetic and sympathetic responses, but not in accordance with the claims of TMP.

## Data Availability

The datasets used and analyzed during the current study are available from the corresponding author on reasonable request.
